# Zinc intake, microRNA dysregulation, and esophageal cancer

**DOI:** 10.18632/aging.100978

**Published:** 2016-06-08

**Authors:** Louise Y. Fong, John L. Farber, Carlo M. Croce

**Affiliations:** Department of Pathology, Anatomy & Cell Biology, Sidney Kimmel Cancer Center, Thomas Jefferson University, Philadelphia, PA 19107, USA

**Keywords:** dietary zinc intake, microRNA dysregulation, esophageal squamous cell carcinoma, dose-response

Esophageal squamous cell carcinoma (ESCC) is a deadly disease with a 5-year survival of only 10% [1]. Because of the absence of early symptoms, ESCC is commonly diagnosed at an advanced stage. Thus, the development of new prevention and therapeutic approaches are urgently needed. Dietary zinc (Zn) deficiency (ZD), a significant public health issue affecting 31% of the global population [2], is associated with an increased risk of ESCC development [3]. Since marginal Zn deficiency is prevalent in humans [4], it was important to learn the effect of mild to moderate Zn deficiency on ESCC etiology.

Our previous work, using rat model systems, has advanced understanding of the role of Zn in ESCC carcinogenesis. Zn deficiency promotes ESCC by inducing an inflammatory gene signature with up-regulation of cancer-associated inflammation genes & an oncogenic microRNA (miRNA) signature featuring up-regulation of oncogenic miR-31 [5]. Our recent dose-response study [6] showed that miRNA dysregulation and esophageal cancer development depended on the extent of dietary Zn deficiency. This tumorigenesis study used low doses of the environmental carcinogen *N*-nitrosomethyl-benzylamine (NMBA) in rats fed diets containing different amounts of Zn (3, 6, 12, or 60 mg Zn/kg) to represent marked-ZD, moderate-ZD, mild-ZD, and Zn-sufficiency in human Zn nutrition. miRNA expression profiling of human tumors has identified signatures associated with staging, progression, prognosis, and response to treatment [7]. We correlated miRNA expression changes with the above noted ZD doses and ZD-associated esophageal tumor outcome by performing miRNA profiling of esophageal mucosa from carcinogen-treated and untreated rats at study endpoint. Marked zinc deficiency (3 mg Zn/kg diet) alone induced a highly proliferative/inflammatory esophagus accompanied by an oncogenic 5-microRNA signature (miR-31, -223, -21, -146b, -146a). Moderate & mild zinc deficiency (6 & 12 mg Zn/kg diet) also induced sustained hyperplasia and inflammation, albeit less pronounced than marked deficiency, with a 2-microRNA signature (miR-31, -146a). The oncomiR miR-21 that was prominently overexpressed (up 4.2 fold) in the highly hyperplastic marked zinc deficient esophagus, however, was not differentially expressed in the less hyperplastic moderate/mild zinc deficient esophagus, providing evidence that miRNA signatures distinguish the highly hyperplastic esophageal phenotype induced by marked deficiency from the less hyperplastic esophageal phenotype induced by moderate and mild zinc deficiency.

With exposure to NMBA, ∼16% of moderate/mild-ZD rats developed ESCC, an incidence greater than for Zn-sufficient rats (0%) (*P*≤0.05) but lower than for marked-ZD rats (68%) (*P<*0.001) (n=27 rats/cohort). These data show a dose-response relationship between the extent of zinc deficiency and ESCC development and provide the first evidence that moderate to mild zinc deficiency, combined with low doses of the environmental carcinogen NMBA, produces ESCC. Importantly, the high ESCC-burden, marked zinc deficient esophagus had a 15-miRNA signature, with strong to modest up-regulation of oncogenic miR-223, -21, -31, -146a, -146b, -27a, -221, -27b, -194, -24, -203, -183, -130b, -106b, -22 (up 3.6 to 1.4 fold), that resembled the human ESCC miRNAome. By contrast, low ESCC-burden, moderate & mild-ZD esophagus displayed, respectively, a 3-miRNA signature (miR-223, -31, -27b) and a 2-miRNA signature (miR-223, -31) with modest up-regulation. Thus, miRNA-signatures can distinguish the divergent ESCC progression in marked zinc deficiency *versus* moderate/mild zinc deficiency rat cohorts, as well as discriminate the hyperplastic marked deficiency esophageal phenotype from the less hyperplastic moderate/mild deficiency phenotype. Together, the data showed that these miRNA signatures not only differentiate stages of ESCC initiation and progression but they also highlight the molecular impact of dietary zinc deficiency on miRNA dysregulation in the pathogenesis of ESCC. In addition, *Fbxw7, Pdcd4*, and *Stk40* (tumor-suppressor targets of miR-223, miR-21 and miR-31, respectively) were down-regulated in the markedly deficient cohort at the mRNA and protein level and they were predicted to interact to alter network of target proteins in cancer pathways. The data are in accord with the finding that miR-223, miR-21 and miR-31 expression levels play important roles in ESCC and may be useful prognostic biomarkers and therapeutic targets for ESCC. Using ChIP-seq and an antibody for histone mark H3K4me3, we previously showed that in Zn-deficient esophagus, the miR-31 promoter region and NF-κB binding site were activated [5]. However, the mechanism(s) by which miR-223 and miR-21 are upregulated by zinc deficiency remains to be elucidated.

In summary, our recent work [6] showed for the first time that ESCC development and the underlying miRNA dysregulation are dependent on the extent of dietary deficiency of the nutrient Zn. The data have identified Zn deficiency-associated miRNA signatures that may underlie the molecular pathogenesis of ESCC in Zn-deficient populations. These findings suggest that dietary Zn may have preventive and therapeutic properties for ESCC.
